# Natural Radioactivity Levels of the Beach Sands of Cleopatra Beach and Damlatas Beach (Türkiye) and Their Impact on Human Health

**DOI:** 10.1021/acsomega.3c05555

**Published:** 2023-12-27

**Authors:** Sezer Unal, Mustafa Gurhan Yalcin, Sema Bilge Ocak

**Affiliations:** † Engineering Geology, Akdeniz University, 07058 Antalya, Türkiye; ‡ Department of Advanced Technologies, Graduate School of Natural and Applied Sciences, Gazi University, 06500 Ankara, Türkiye

## Abstract

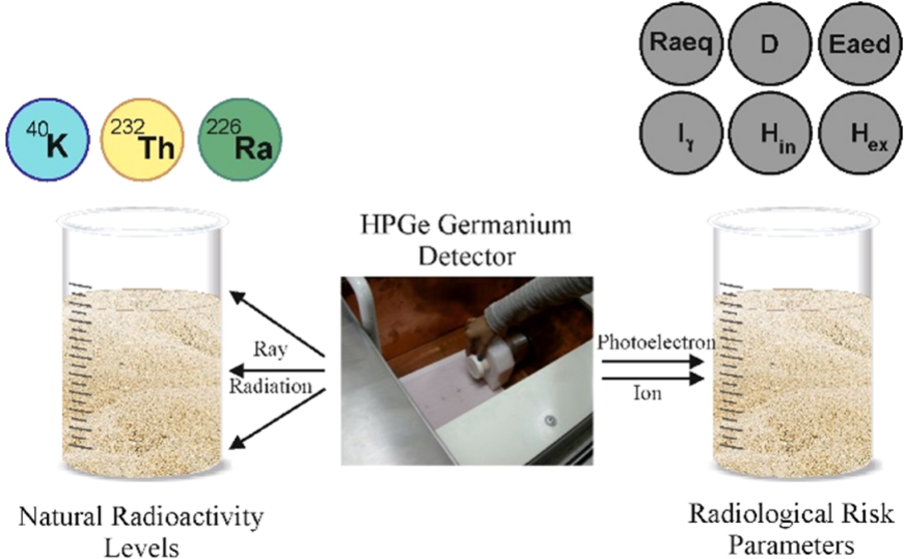

HPGe γ spectrometry method was used to measure the natural radioactivity levels (^40^K, ^232^Th, and ^226^Ra) of the beach sand samples from Cleopatra Beach and Damlatas Beach in Antalya (Türkiye). The mean ^40^K, ^232^Th, and ^226^Ra radioactivity levels of the studied samples were calculated as 276.88 ± 17.24, 25.04 ± 2.88, and 17.06 ± 1.68 Bq/kg, respectively. Being below the radiation limits, these values indicate no risk in terms of public health. Moreover, the radiological risk parameters, such as excess lifetime cancer risk, radium equivalent activity (Ra_eq_), absorbed γ dose rate (D), annual effective dose equivalent (Ea_ed_), γ index (I_γ_), internal radiation hazard index (H_in_), and external radiation hazard index (H_ex_), were calculated. The values of all of these parameters were found to be below the internationally accepted radiation limit values. In addition, distribution maps showing the radiological situation of the region were generated although they did not pose a hazard to public health. No results were found in the analysis processes related to artificial radioactivity.

## Introduction

The environment where people live is naturally radioactive. Checking radioactivity levels is very important because these values are used to evaluate the dose emitted in places where people live as well as to determine the basic levels of environmental radioactivity for detecting any changes in it.^1^ It is observed that radionuclides are exposed to different amounts of ionizing radiation due to radioactive materials generated by human activities. People’s exposure to radioactivity concentrations depends on the region they live in, as well as the concentration of elements and gases in the natural structure of that region.^2^ Therefore, people are exposed to ionizing radiation due to radionuclides that naturally occur every day.^3^ All living organisms in the universe are composed of a kind of radioactive materials and the materials are affected by the different radiation dose.^4−6^ Various geological formations, including some kinds of soils and rocks used in the construction of buildings, may contain materials that contain radionuclides of natural origin.^7^ Radioactive materials contain original terrestrial radionuclides, also known as primitive radionuclides, since the formation of the world, and they are present in various concentrations in the environment. Radioactive materials typically have a long life, and the half-life of these materials is hundreds of millions of years.

Regional enrichments of natural radioactivity are observed in beach sands all over the world. As mineral deposits, beach sands are usually composed of volcanic rocks such as granite, andesite, and rhyolite. Moreover, these rocks might be rich in minerals containing the electrons of Th and U. As a result of the weathering of the rocks because of erosion, these minerals move due to weather conditions and deposit and enrich on the beaches. The radiation doses of the beach sands with higher concentrations of thorium and uranium, are quite high.^8^ The main sources of beach sands are metamorphic and magmatic rocks. Some metamorphic and magmatic rocks contain natural radionuclides that emit radiation into the environment. The elements oxygen, potassium, magnesium, calcium, sodium, silicon, aluminum, and iron constitute about 99% of the crust of the Earth. Low-density minerals, which are the dominant compounds of silicate and aluminosilicates making up about 80% of the earth’s crust, are formed by such elements. While ultramafic rocks might contain higher proportions of titanium, vanadium, iron, and manganese, granite rocks might contain higher concentrations of tungsten, molybdenum, tin, thorium, uranium, and titanium. On the other hand, acidic volcanic rocks might contain higher amounts of copper, zinc, lead, cadmium, and mercury, and shale rocks might contain higher concentrations of lead, cadmium, vanadium, and uranium.^9^

Beach sand samples’ radionuclide levels are very important since they indicate significant details to keep as reference records for determining the possible changes in environmental radioactivity.^10^ Dose rates are common in the world, and they vary from place to place in several geological formations such as air, water, sand, and soil in different concentrations depending on their geological and geographical conditions.^11−14^ Beach sands, which contain coarse materials rich in inorganic silicon, are the weather-resistant residues of geological formations moved from their original places through movement mechanisms such as rivers, winds, and glaciers, and they deposit on the beach by the movements of currents and waves.^15,16^

With a coastline of 8333 km, Türkiye has the longest coastline, as well as the largest beach sand system, in Europe.^17^ Türkiye is rich in beach sands.^18^ The natural radioactivity analysis of the coastline plays a significant role due to this richness. While there are plenty of research on the natural radioactivity levels of the coastline and their doses in various countries and regions of the world, very few studies have been conducted in Türkiye.^19,20^

The present study was carried out to reveal the natural radioactivity levels of the beach sands of Cleopatra Beach and Damlatas Beach, to compute radiological parameters of excess lifetime cancer risk, radiation risk, radium equivalent activity, dose rates, and annual gonadal dose equivalent. The study also aims to interpret the impacts of these beaches on human health by considering the results and the average values reported for other places. All radiological parameters were listed, and their distribution maps were generated by considering the values of all radiological parameters.

## Materials and Methods

### Study Area and Sampling

Antalya province, which lies between the 29°20′-32°35′ East longitudes and between 36°07′-37°29′ North latitudes, is in the southwest of Türkiye. The Mediterranean Sea surrounds Antalya in the south, and the Taurus Mountains lie parallel to the sea in the North of the province. Antalya is surrounded by the provinces of Karaman, Icel, and Konya in the east, Mugla in the west, and Burdur and Isparta in the north. The surface area of the Antalya province is about 20,815 km^2^. This corresponds to 2.6% of the surface area of Türkiye. Antalya province, which is located in the Mediterranean Region, covers 17.6% of the total area of the region.^21^ The length of the coasts of Antalya, which is the most important tourism center of Türkiye, is 640 km, including the recesses and the ledges, while the length of the straight coastline is 500 km.^21^ Since the mountains lie perpendicular to the sea, the sea is deep, and the beaches are not continuous on the western coasts. With its turquoise sea and long coastline of about 70 km, Alanya is one of the most preferred holiday resorts in Antalya. Alanya is located in the Gulf of Antalya, along the southern coast of Anatolia, and in the Pamphylia Plain. It is located within the Turkish Riviera, surrounded by the Mediterranean Sea and the Taurus Mountains in the South and North, respectively.^21^

**Figure 1:**
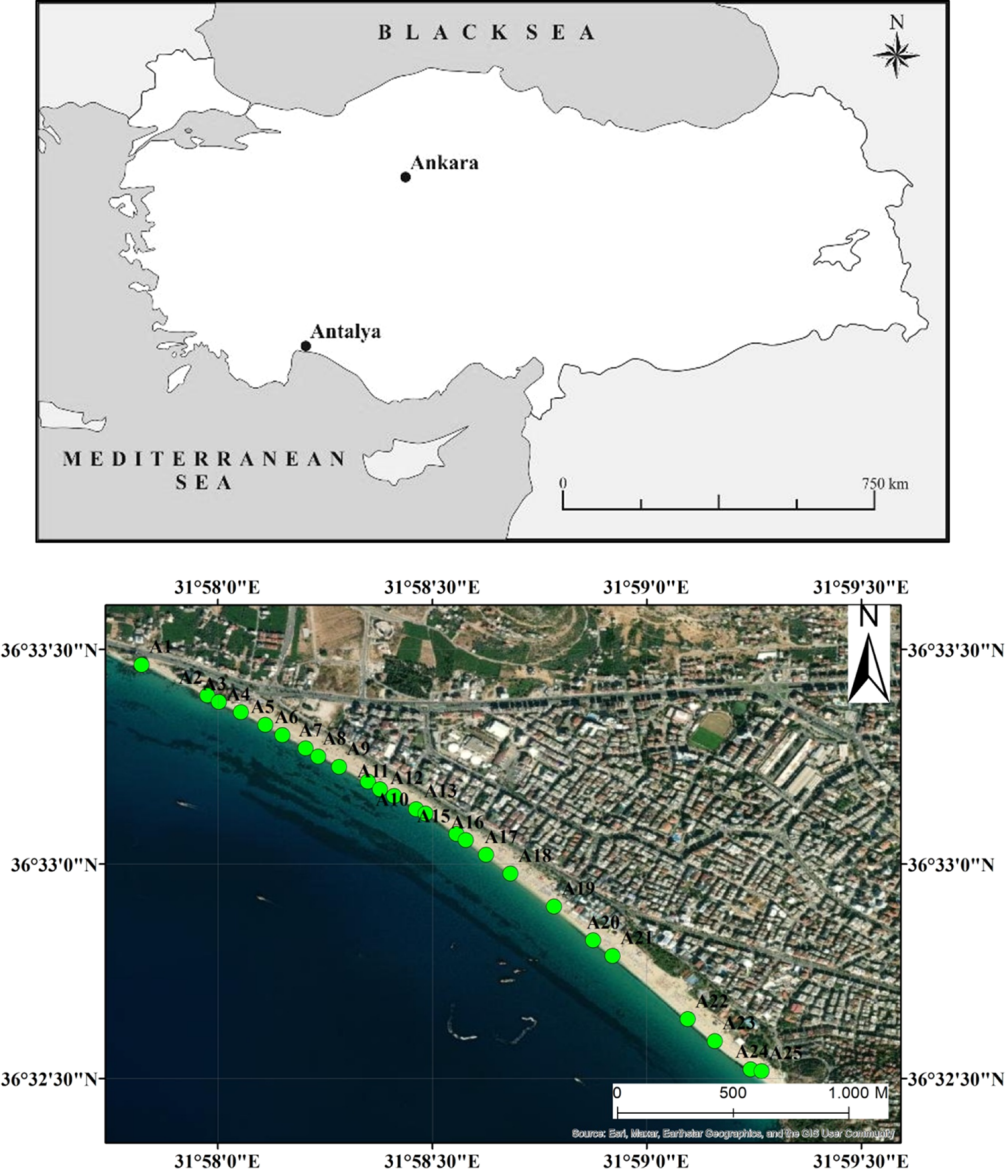
Site location map of the samples collected.^22^

The samples were systematically collected every 50–100 m from Cleopatra Beach and Damlatas Beach in the Alanya district, which is one of the most popular tourism centers of Türkiye, to conduct measurements for radiation analysis. The site location map shows the locations of 25 samples collected (Figure 1).^22^ Similar works were done by Sathish and Chandrasekaran.^23^ The rapid increase in population and the growth of the tourism industry in recent years have increased the demand for beaches. Heavy metal accumulation on coastlines, which host millions of people and terrestrial organisms every year, has become an important environmental problem.

### Regional Geology

The southern section of the Central Taurus Mountains covers the location of the study area. In this region, there are sedimentary rock assemblages and ultrabasic rocks with different ages and lithologies and regular or irregular stratigraphic characteristics that also reflect the ambient conditions in a different way. The relationship between these sedimentary rocks and ultrabasic rocks can be seen in six central units, as they are tectonic. From the north to the south end, these central units cover the Aladag unit, Antalya unit, Pirnos-Tepedag unit, Alanya unit, Paleo-autochthonous cover rocks, and neo-autochthonous cover rocks covering previously formed units with incompatibility; most importantly, these central units are of Upper Oligocene (?) - Miocene ages.^24^

The Alanya massif, located to the east of the Gulf of Antalya, consists of Paleozoic-Mesozoic and a Precambrian-aged metasedimentary cover, which is thought to be an alternative to the Pan-African foundation of the Gondwana plate. Alanya and Antalya units are reported to be the stratigraphic and structural continuation of other units. The structure of the Alanya massif generally includes Paleozoic-aged units. An overturned anticlinorium has been formed toward the south, while a subduction synclinorium has been formed toward the southeast, mostly composed of Mesozoic-aged units. Since the Alanya massif here is due to a northward sloping tectonic slicing and a spatially progressive Alpine-aged degradation process, it is reported as the combined result of the disappearance of the Paleotethys plate toward the south on the Anatolian-Apulia microcontinent and toward the north of the Tethys plate. The decline of the degradation effect toward the northeast depends on the fact that it is inclined toward the upper structural positions in this direction as it moves away from the supra-subduction zone. The upper Cretaceous-Eocene-aged sedimentary units on the Alanya and Antalya units are formed in motion.^24^

### HPGe γ Spectrometry

The net peak areas with the least error have been calculated for each peak sample detected using a high-purity germanium (HPGe) detector with high-resolution γ spectroscopy. Radioactivity measurement was conducted by utilizing an N-type coaxial HPGe detector cooled by liquid nitrogen and with a −5000 V negative bias voltage γ-ray detector made by Mirion Technologies (EGC 100–240-R S/N). The detector’s relative efficiency is 100%, and it has a resolution of 1.04 keV full width at half-maximum (fwhm) at 122 keV for ^57^Co and 2.39 keV fwhm at 1332 keV for ^60^Co. It is connected to CAEN-Hexagon consisting of an HV supply and Preamplifier Power Supply, fast analog-to-digital converter (ADC), dual digital 32k multi-channel analyzer (MCA), which integrates the input stage for the signal conditioning, and digital processing algorithms in a compact desktop form factor. After the radioactivity calibration coefficients were determined in the spectrum, they were transferred to the main center of the computer. Then, the energy activation values were determined. The radioactivity series of ^232^Th and ^238^U (^226^Ra) and the γ energy values received for ^40^K were shown. For the series of ^238^U (^226^Ra), the areas of the peak region for the energies of ^214^Bi (609.4 keV), ^214^Pb (352.0 keV), and ^214^Pb (295.2 keV) were obtained; for the series of ^232^Th, the areas of the peak region for the γ energies of ^228^Ac (911.1 keV), ^208^Tl (583.1 keV), and ^212^Pb (238.6 keV) were obtained; for ^40^K, the area of the peak at the γ energy of (1460.8 keV) was obtained..^25^

### Dose Calculations

After drying the beach sand samples, each sample was ground for abound 10 min using the mortar grinder RM 200 (Retsch GmbH, Germany). After the samples were ground, a 2 mm sieve was used to sieve each sample for carrying out the sieve analysis. The samples were placed in 50 cc sample containers that were homogenized by washing with distilled water. Then, the tare weight was determined, each sample was weighed in grams by using a precision scale, and the weights were recorded.

After these procedures, the covers of the sample containers were tightly closed by using insulating tape so that they could be airtight. After closing the sample containers, all samples were kept in a place without sunlight for about one month to determine the decay equation between ^226^Ra and ^222^Rn and to allow the stabilization of the Compton field. The samples, which were ready for measurements after one month, were sent to the Turkish Accelerator and Radiation Laboratory for analysis by utilizing an HPGe detector. In this laboratory, each of the samples was analyzed with a counting time of about 50,000 s. According to the results of the count analysis, the peak areas with radionuclides were determined and the activity values of each sample were calculated and recorded. High-resolution γ spectroscopy was determined to give the clearest peak areas with the least error for all peak samples in the spectrum.

The ^226^Ra and ^232^Th radioactivity concentrations and the ^238^U and ^232^Th radioactivity decay series of the samples were determined in the γ spectra using eq 1 as follows
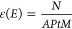
1where *A* denotes the activity of the radionuclide and its unit is Bq kg^–1^, *N* denotes the total net count in the γ energy, *t* denotes the counting time (s), ε(*E*) denotes the detector efficiency at energy *E*, and *M* is the mass of the sample. Finally, *P* denotes the absolute transition probability of γ-decay of the nuclide at energy *E*.^23^

### Radium Equivalent Activity (Ra_eq_)

Besides being used as a construction material, beach sands are used in various industrial Radioactivity levels of beach sands, similar to those of other environmental matrices, are usually found using the contents of the radioactivity elements of ^40^K, ^232^Th, and ^226^Ra, which are not spread uniformly. If a material contains K, Th, and Ra, its nonuniform radioactivity is determined by employing the widely used radium equivalent activity index (Ra_eq_). The Ra_eq_ index, which indicates the specific activities of ^40^K, ^232^Th, and ^226^Ra in just one value, considers the radiation risks caused by each of these radionuclides.^26−29^ A_K_, A_Th_, and A_Ra_, which stand for the ^40^K, ^232^Th, and ^226^Ra activity concentrations, respectively, can be obtained using eq 2 as follows
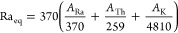
2Being the weighted sum of three specific radionuclides’ activities, the value of Ra_eq_ first depends on external and internal γ doses, and second, depends on radon and its progenies. 259 Bq kg^–1^ of ^232^Th, or 370 Bq kg^–1^ of ^226^Ra, or a quantity of ^40^K that generates an equal value of γ dose rate generates 4810 Bq kg^–1^^26−29^

### Absorbed γ Dose Rate (D) in Beach Sands

The absorbed dose rate by air due to γ radiation from terrestrial radionuclides is widely used to estimate external exposure in beach sands, and it is defined as the value of energy due to ionizing radiations absorbed per unit time per unit air mass.

In the event that the beach sand’s radionuclide activity is known, its exposure dose rate in the outside air at 1 m above the ground level due to this radionuclide activity is computed using eq 3(26−29)
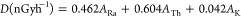
3where the coefficients of 0.042, 0.604, and 0.462 are the dose conversion factors, and they convert the ^40^K, ^232^Th, and ^226^Ra activity concentrations into dose rates (in nGyh^–1^ per Bq kg^–1^), respectively.^12^

### Annual Effective Dose Equivalent (Ea_ed_) of the Beach Sands

People working around the beach and people who have settled in the places near the beach area are those who were greatly affected by the radiation dose due to the beach. The Ea_ed_ value is estimated using *D* by the air by using the conversion factor 0.7 SvGy^–1^, which is applied to the effective dose taken by an adult individual, and the outdoor occupancy factor 0.2.^12^ Finally, the Ea_ed_ value for an individual is calculated in mSv y^–1^ by using eq 4 as follows^26−31^
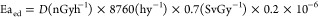
4

### γ Index (Iγ) Levels of the Beach Sands

The γ index (Iγ) levels are considered to assess the γ-radiation hazard due to natural γ emitters contained by the beach sand. γ index, which associates excessive γ-radiation from superficial materials with the annual dose rate, can be used as a screening tool in the classification of materials if they are used in the construction of a building.^26^ The γ index (Iγ) is calculated using eq 5.^32^
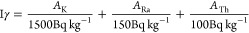
5

### Internal Hazard Index (H_in_) and External Hazard Index (H_ex_) of the Beach Sands

H_in_ and H_ex_ values of the construction materials are determined for the requirement of keeping the radiation dose within a permissible limit, that is, below 1 mSv y^–1^.^26−32^ The H_ex_ value is calculated by employing eq 6.^26−34^
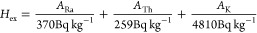
6

Moreover, inhaling α-emitting radionuclides ^220^Rn (^224^Ra’s generation) and ^222^Rn (^226^Ra’s generation) leads to health risks to the respiratory tract. The H_in_ value is utilized to calculate the exposure to Rn and Tn and their products as follows:^25−31^
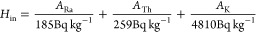
7

### Excess Lifetime Cancer Risk (ELCR) of Beach Sands

ELCR is a very significant radiological parameter because it is utilized to evaluate the risk of developing excessive cancer due to radiation exposure throughout an individual’s life.^27^ The ELCR value of beach sands was calculated using Ea_ed_, *A*_lf_, and *R*_f_ in eq 8.^32^
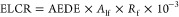
8

The ELCR value is calculated by multiplying AEDE, mean life expectancy (70 years) (*A*_lf_), fatal cancer risk factor (*R*_f_), and 10^–3^. The value of ELCR should be below the world average (0.29 × 10^–3^). A value of 0.05 per Sievert is reported as a risk factor by the ICRP Publication 60 due to stochastic effects.^27−32^

## Results and Discussion

**Figure 2:**
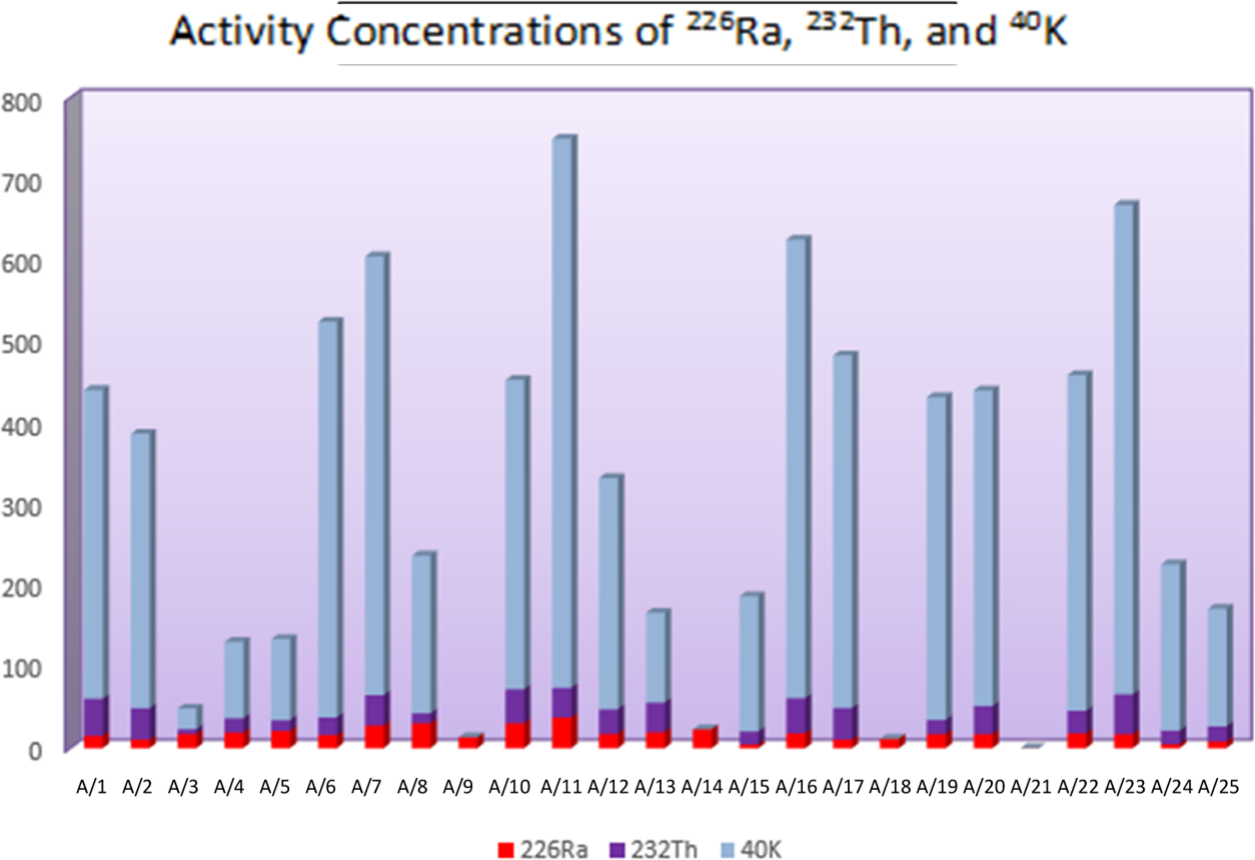
^226^Ra, ^232^Th, and ^40^K activity concentrations of the samples.

The U-238 (Ra), K-40, and Th-232 activity values were calculated for 25 beach sand samples collected from Cleopatra Beach and Damlatas Beach in Türkiye (Table 1 and Figure 2).

**Table 1:** Radionuclide Activity Concentrations and Radiological Parameters of Samples from Cleopatra and Damlatas Beach

sample	coordinates in DMS (N,E)		^226^Ra	^232^Th	^40^K	Ra_eq_	*D*	AEDE	Iγ	H_ex_	H_in_	ELCR × 10^–3^
*A*/1	36°33′27″	31°57′49″	14.96	**45.84**	379.8	109.66	50.55	0.06	0.81	0.3	0.34	0.22
*A*/2	36°33′23″	31°57′58″	10.46	**38.73**	337.56	91.75	42.4	0.05	0.68	0.25	0.28	0.18
*A*/3	36°33′22″	31°57′59″	17.52	5.94	25.2	27.94	12.74	0.02	0.19	0.07	0.12	0.05
*A*/4	36°33′21″	31°58′03″	19.11	17.63	94	51.52	23.42	0.03	0.36	0.14	0.19	0.1
*A*/5	36°33′19″	31°58′06″	21.24	13.19	99.83	47.75	21.97	0.03	0.34	0.13	0.19	0.09
*A*/6	36°33′18″	31°58′09″	15.97	21.84	**486.77**	84.62	41.02	0.05	0.65	0.23	0.27	0.18
*A*/7	36°33′16″	31°58′12″	27.71	**37.45**	**539.83**	122.73	**58.09**	0.07	0.92	0.33	0.41	0.25
*A*/8	36°33′15″	31°58′14″	30.51	12.31	194.16	63.03	29.68	0.03	0.46	0.17	0.25	0.13
*A*/9	36°33′13″	31°58′16″	13.5	BLD	BLD	13.5	6.24	BLD	0.09	0.04	0.07	0.03
*A*/10	36°33′11″	31°58′20″	30.57	**41.98**	380.49	119.81	**55.46**	0.07	0.88	0.32	0.41	0.24
*A*/11	36°33′10″	31°58′22″	**37.61**	**36.55**	**675.48**	141.78	**67.82**	0.08	**1.07**	0.38	0.48	**0.29**
*A*/12	36°33′09″	31°58′24″	17.6	**30.23**	284.47	82.68	38.34	0.05	0.61	0.22	0.27	0.16
*A*/13	36°33′07″	31°58′27″	19.66	**36.35**	110.44	80.08	35.67	0.04	0.57	0.22	0.27	0.15
*A*/14	36°33′07″	31°58′29″	23.09	BLD	BLD	23.08	10.66	0.01	0.15	0.06	0.12	0.04
*A*/15	36°33′04″	31°58′33″	4.36	16.11	166.44	40.17	18.73	0.02	0.3	0.11	0.12	0.08
*A*/16	36°33′03″	31°58′34″	18.09	**43.37**	**564.16**	123.44	**58.24**	0.07	0.93	0.33	0.38	0.25
*A*/17	36°33′01″	31°58′37″	10.52	**38.81**	**433.72**	99.32	46.51	0.06	0.75	0.27	0.3	0.2
*A*/18	36°32′58″	31°58′40″	11.39	BLD	BLD	11.39	5.26	BLD	0.076	0.03	0.06	0.02
*A*/19	36°32′54″	31°58′47″	17.05	17.73	397.18	72.92	35.26	0.04	0.56	0.2	0.24	0.15
*A*/20	36°32′49″	31°58′52″	17.21	34.6	388.31	96.51	45.16	0.06	0.72	0.26	0.31	0.19
*A*/21	36°32′47″	31°58′55″	0.29	BLD	BLD	0.29	0.13	BLD	BLD	BLD	BLD	BLD
*A*/22	36°32′38″	31°59′05″	18.01	27.9	**412.92**	89.63	42.51	0.05	0.67	0.24	0.29	0.18
*A*/23	36°32′35″	31°59′09″	17.23	**49.01**	**602.13**	133.56	**62.85**	0.08	**1.01**	0.36	0.41	0.27
*A*/24	36°32′31″	31°59′14″	4.89	16.57	204.68	44.3	20.86	0.03	0.33	0.2	0.13	0.09
*A*/25	36°32′31″	31°59′16″	8.07	18.76	144.57	45.99	21.13	0.03	0.33	0.12	0.15	0.09
min			0.29	BLD	BLD	0.29	0.13	BLD	BLD	BLD	BLD	BLD
max			37.61	49.01	675.48	141.78	67.82	0.08	1.07	0.38	0.48	0.29
UNSCEAR			**35**	**30**	**400**	**370**	**55**	**0.46**	**1**	**<1**	**<1**	**0.29**

According to the Ra_eq_ activity values of the samples from Cleopatra Beach and Damlatas Beach, sample *A*/21 was found to have the lowest activity value (0.29), while sample *A*/11 was found to have the highest activity value (141.78).

According to the *D* values of the samples from Cleopatra Beach and Damlatas Beach, sample *A*/21 was found to have the lowest activity value (0.13), while sample *A*/11 was found to have the highest activity value (67.82). According to the Eaed activity values of the samples from Cleopatra Beach and Damlatas Beach, samples *A*/9, *A*/18, and *A*/21 were found to have the lowest activity value (BLD) while samples *A*/11 and *A*/23 were found to have the highest activity value (0.08). According to Iγ activity values of the samples from Cleopatra Beach and Damlatas Beach, sample *A*/21 was found to have the lowest activity value (BLD) while sample *A*/11 was found to have the highest activity value (1.07). Considering the H_ex_ activity values of the samples from Cleopatra Beach to Damlatas Beach presented in Table 1, sample *A*/21 was found to have the lowest activity value (BLD) while sample *A*/11 was found to have the highest activity value (0.38).

According to the H_in_ activity values of the samples from Cleopatra Beach and Damlatas Beach presented in Table 1, sample *A*/21 was found to have the lowest activity value (BLD) while sample *A*/11 was found to have the highest activity value (0.48). According to the ELCR activity values of the samples from Cleopatra Beach to Damlatas Beach, sample *A*/21 was found to have the lowest activity value (BLD) while sample *A*/11 was found to have the highest activity value (0.29).

**Figure 3:**
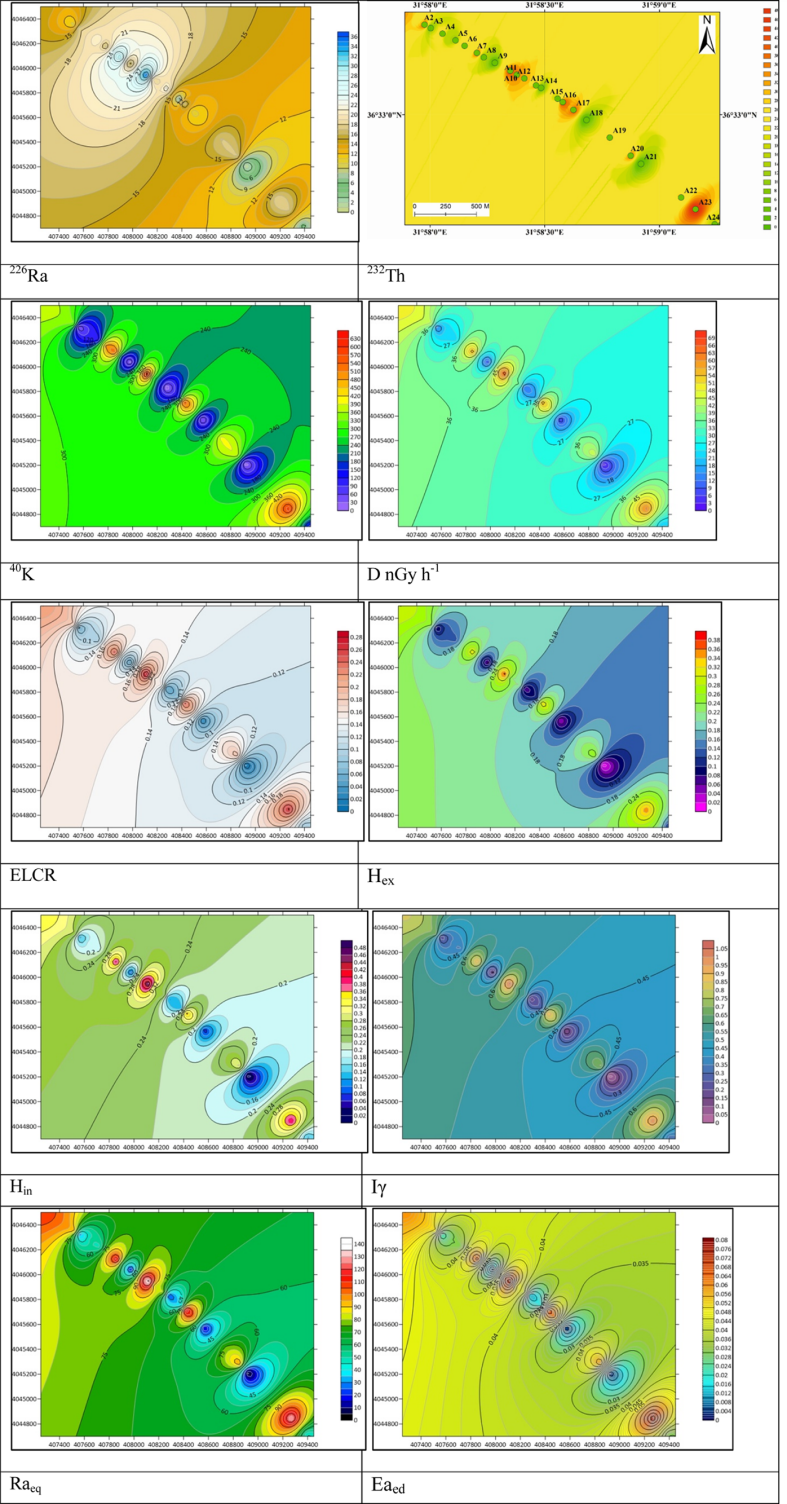
Distribution maps of all radiological parameters of the study area.

As can be seen in Table 2, a comparison of the results of the present study with those of studies conducted in the coastal regions of other countries revealed that the radiation dose levels of the analyzed samples were significantly below the world average (Figure 3).

**Table 2:** Activity Results of Beach Sands from Countries

	activity concentrations (Bq kg^–1^)			
country	^226^Ra	^232^Th	^40^K	reference value
Türkiye	0.29–37.61	BLD– 49.01	BLD–675.48	present study
India—Tamil Nadu	24.35–55.74	22.11–50.11	223.78–320.38	(19)
Southern India	1.56–32.38	34.77–229.30	5.21–88.39	(35)
Malaysia	451.4–2411.4	232.2–1271.9	60.9–135.6	(36)
Ghana	14	30	320	(37)
Omani Sea	14.96	17.61	361.60	(38)
Saudi Arabia	26.4	16.3	351	(39)
Greece	12–2292	16–10.143	191–1192	(30)
Bangladesh	2400–2500	3300–4300	80–260	(40)
China	7.8–25.7	6.5–41.4	197.4–487.6	(41)
India—Kerala	99–3192	1288–18.515		(42)
Montenegro	2.09–15.46	1.37–16.58	7.13–304.87	(43)
Romania	2.9–14.0	1.2–8.5	9–233	(44)
Srilanka	7–1243	14–6257	170–647	(45)
Iran	14.6–29.6	14.8–21.7	179.5–464.5	(46)
global	35	30	400	(12)

**Figure 4:**
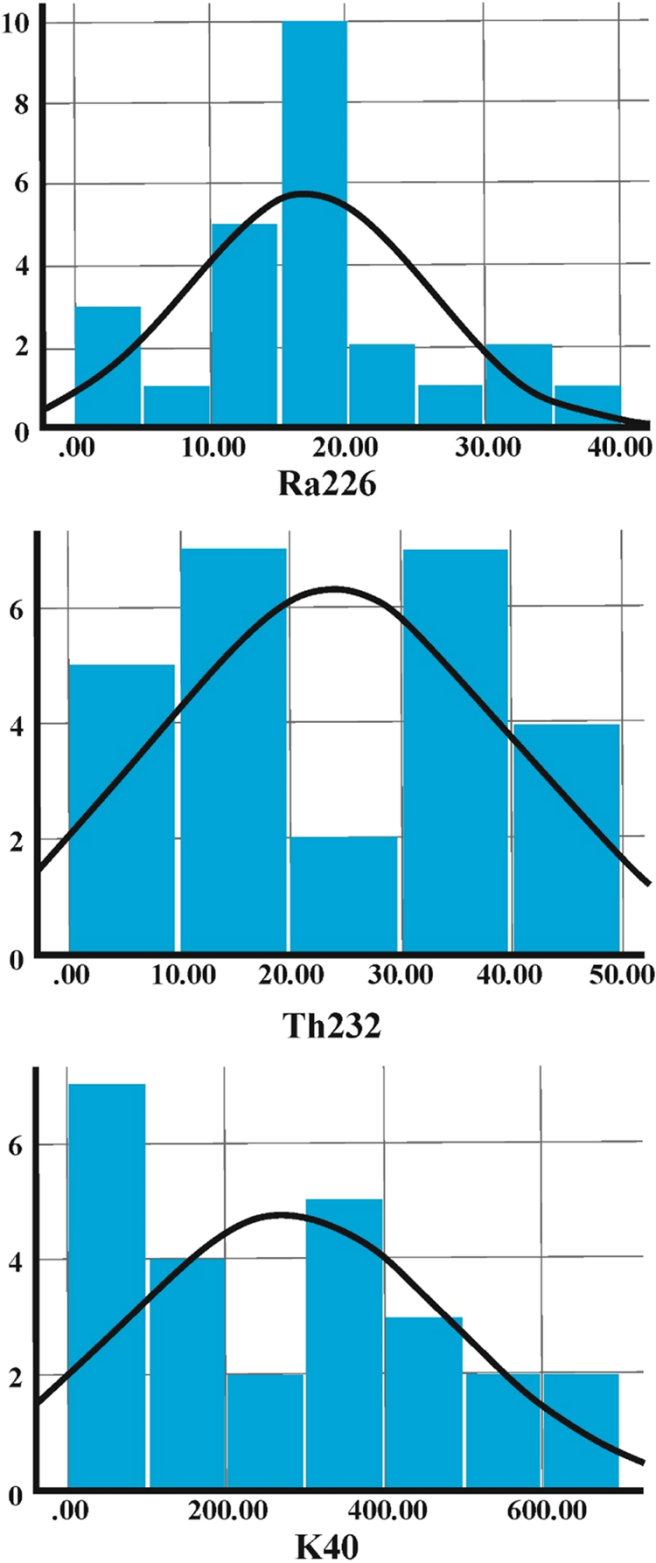
Histograms of the distribution of radionuclides ^226^Ra, ^232^Th, and ^40^K for beach sand samples from Cleopatra Beach and Damlatas Beach.

**Figure 5:**
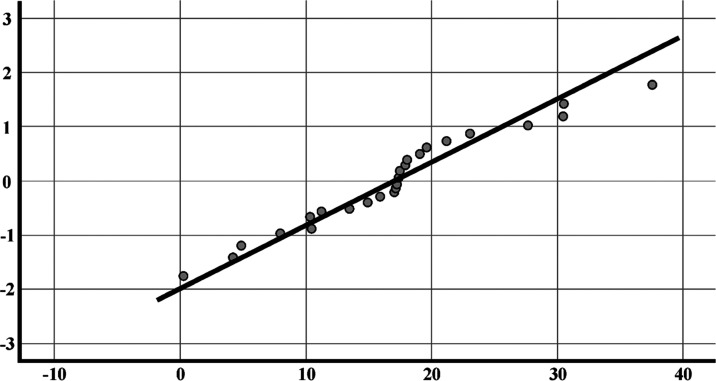
Normal distribution of frequencies of radionuclides ^226^Ra, ^232^Thi, and ^40^K.

In the present study on the beach sands from Cleopatra Beach and Damlataş Beach, the standard deviations of the ^40^K, ^232^Th, and ^226^Ra activity concentrations were found below mean values. However, as seen in Table 2, this can be explained as an indication of a high degree of uniformity. The values of the descriptive statistics such as maximum, minimum, median, average, standard error, standard deviation, variance, kurtosis, and skewness were calculated by using the SPSS 26 software package and are presented in Table 3. Figure 4 revealed that the skewness value for ^232^Th activity concentrations was negative while the skewness value for ^226^Ra and ^40^K were found to be positive but close to zero. The results of the study on Cleopatra Beach to Damlatas Beach reveal that the kurtosis values of the ^232^Th and ^40^K activity concentrations are negative (Table 3). Figure 5 reveals that the frequency distribution has a normal distribution. Another study carried out in the Antalya region reported the following minimum and maximum values: Iα (0.01–0.32), *D* (6.78–33.82), Ra_eq_ (14,12–72.8), H_in_ (0.04–0.2), H_ex_ (0.05–0.37), AGDE (48.06–228.7), AEDE (8.32–41.48), and ELCR outdoor (0.03–0.15),^12^ and these values were found below the limit values.^46^ The analysis revealed that the radioactivity values varied between 1 and 104 Bq kg^–1^ for ^232^Th, 0 and 212 Bq kg^–1^ for ^238^U (Ra), and 29 and 986 Bq kg^–1^ for the ^40^K activities.^47,48^ Since the radioactivity levels of some beaches in Tekirova, Kemer, and Kumluca were reported to be high, people living in these areas should have a health check-up. The concentrations of the radioactive elements of thorium (^232^Th), uranium (^238^U), and potassium (^40^K) in the natural rocks in the vicinity of Antalya province were calculated using a high-purity germanium (HPGe) detector with γ spectrometry as 12.64, 20.22, and 238.49 Bq kg^–1^, respectively.^49^

**Table 3:** Descriptive Statistics for the Radionuclide and Radiological Parameters of the Beach Sand Samples from Cleopatra Beach and Damlatas Beach

	radiological parameters									
statistical variables	^226^Ra	^232^Th	^40^K	Ra_eq_	*D* nGyh^–1^	Ea_ed_	Iγ	H_ex_	H_in_	ELCR
minimum	0.29	BLD	BLD	0.29	0.13	BLD	BLD	BLD	BLD	BLD
maximum	37.61	49.01	675.48	141.78	67.82	0.08	1.07	0.38	0.48	0.29
mean	17.06	24.03	276.88	72.69	34.02	0.04	0.53	0.19	0.24	0.14
median	17.23	21.84	284.47	80.08	35.67	0.04	0.57	0.22	0.27	0.15
standard deviation	8.60	15.82	210.12	40.86	19.32	0.02	0.31	0.10	0.12	0.08
standard error	1.71	3.16	42.02	8.17	3.86	BLD	0.06	0.02	0.02	0.016
variance	73.94	250.32	44154.28	1669.80	373.42	BLD	0.09	0.01	0.01	BLD
skewness	0.355	–0.147	0.191	–0.079	–0.048	–0.169	–0.056	–0.183	–0.038	–0.04
kurtosis	0.475	–1.251	–1.152	–1.033	–1.011	–0.90	–1.055	–0.948	–0.716	–1.035
frequency distribution	normal	normal	normal							

The distribution map of natural radioactivity levels of the samples from Cleopatra Beach and Damlatas Beach was generated using Surfer 21.1.158 software. The distribution maps of all radiological parameters were generated and are presented in Figure 3.

## Conclusions

A total of 25 samples were collected from Cleopatra Beach and Damlatas Beach in Antalya province, and the activity concentrations of ^226^Ra, ^232^Th, and ^40^K, which are natural radionuclides, were measured for these samples. While the ^226^Ra activity concentration was observed to range between 0.29 and 37.61 Bq kg^–1^, only one sample’s (*A*/11) ^226^Ra activity concentration was found to exceed the average limit value (35 Bq kg^–1^) specified by the UNSCEAR Report. While the ^232^Th activity concentration was found to range between BLD and 49.01 Bq kg^–1^, samples *A*/1, *A*/2, *A*/7, *A*/10, *A*/11, *A*/13, *A*/16, *A*/17, *A*/20, and *A*/23 were observed to exceed the average limit value (30 Bq kg^–1^) specified by the UNSCEAR Report in terms of ^232^Th activity concentration. While the ^40^K activity concentration ranged between 0.00 and 675.48 Bq kg^–1^, samples *A*/5, *A*/7, *A*/11, *A*/16, *A*/17, and *A*/23 were observed to exceed the average limit value (400 Bq kg^–1^) specified by the UNSCEAR Report in terms of ^40^K activity concentration.

The samples collected from Cleopatra Beach and Damlatas Beach in Antalya province were analyzed to determine their radiological parameters. While the Ra_eq_ values of the samples ranged between 0.29 and 141.78, the values of all samples were lower than the limit value (370 Bq kg^–1^) specified by the UNSCEAR Report. The activity values of the absorbed γ dose rate were found to vary between 0.13 and 67.82 nGyh^–1^. The absorbed γ dose rate activity values of samples *A*/7, *A*/10, *A*/11, *A*/16, and *A*/23 were found to exceed the limit value (55 nGyh^–1^) specified by the UNSCEAR Report. It was found that the value of the annual effective dose equivalent (Ea_ed_) varied between BLD and 0.08 mSv y^–1^, and the Ea_ed_ values of all samples were found to be below 0.46 mSv y^–1^, which is specified as the limit value by the UNSCEAR Report. The γ index value was found to range between BLD and 1.07 Iγ, and the γ index values of samples *A*/11 and *A*/23 were found to exceed the limit value (1 Iγ) specified by the UNSCEAR Report. It was also found that the activity values of Hex and Hin were lower than 1 Sv y^–1^, which is specified as the limit value by the UNSCEAR Report. Thus, they were observed to pose no radiological hazards. The ELCR values of all samples were found to be below 0.29 × 10^–3^, which is specified as the limit value by the UNSCEAR Report, indicating that the studied samples pose no radiological hazards.
